# Branched-chain amino acid transaminases as promising targets in tumor therapy

**DOI:** 10.3389/fcell.2026.1712076

**Published:** 2026-02-04

**Authors:** Weiran Zhang, Jie Shen, Xuanyin Ding, Hele Liu, Xu Wang, Dan Feng

**Affiliations:** 1 Department of Thoracic Oncology, Cancer Institute of Jiangsu University, Affiliated Hospital of Jiangsu University, Zhenjiang, Jiangsu, China; 2 Department of Pharmacy, Affiliated Hospital of Jiangsu University, Zhenjiang, China

**Keywords:** biomarker, branched-chain amino acid transaminases, drug resistance, oncogenic pathway, tumor metabolism

## Abstract

Branched-chain amino acid transaminases (BCATs), including BCAT1 and BCAT2, play pivotal roles in tumorigenesis and therapeutic resistance in various cancers. These enzymes regulate branched-chain amino acid (BCAA) metabolism and influence critical oncogenic pathways such as mTOR, PI3K/AKT, and Wnt/β-catenin signalling. Furthermore, BCATs contribute to metabolic reprogramming, epigenetic modifications, and immune evasion. Collectively, they promote tumor proliferation, invasion, and metastasis, thus making BCATs potential biomarkers and therapeutic targets. Recent studies highlight their aberrant expression in cancers including gastric cancer, pancreatic cancer, non-small cell lung cancer, leukaemia, gliomas, and breast cancer, where they contribute to resistance to chemotherapy, targeted therapy, and endocrine therapy. Strategies targeting BCATs, including enzyme inhibitors, dietary BCAA restriction, and combination therapies, have shown the potential to overcome drug resistance and improve treatment outcomes. This review synthesizes current knowledge on the mechanisms of BCATs in cancer progression and resistance, providing a foundation for future research and clinical applications.

## 1 Introduction

Branched-chain amino acids (BCAAs) are essential amino acids, including leucine, isoleucine and valine, all of which have branched side chains. These three amino acids not only serve as building blocks for proteins but also play roles in cell signalling, energy use and storage, and immune system function (Bonvini et al., 2018; Ericksen and Han, 2019). BCAAs provide fuel for cancer cells, activate the mTOR pathway, stimulate protein synthesis, and promote cell division. Furthermore, BCAAs promote tumor cell development by regulating glucose metabolism. Crucially, by impairing CD8^+^ T-cell effector function and weakening antitumor immunity, BCAAs assist tumor cells in evading immune surveillance (Yao et al., 2023).

**FIGURE 1:**
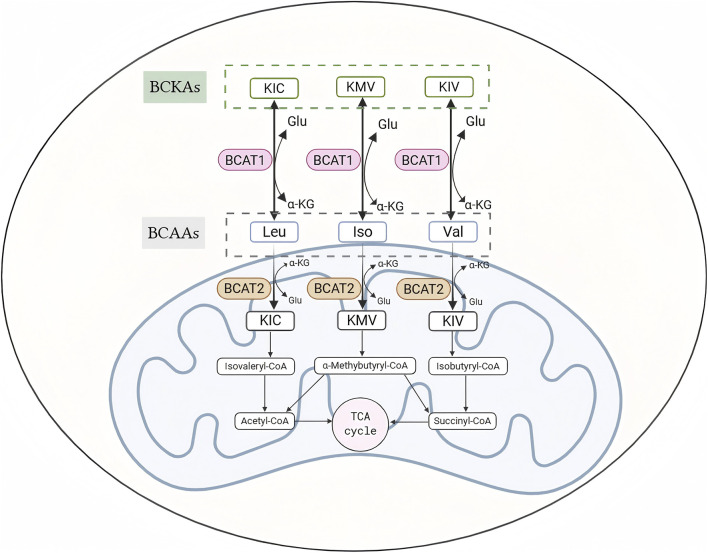
BCAA Catabolism BCATs (BCAT1 and BCAT2) transfer the α-amino group from BCAAs (leucine, isoleucine, valine) to α-ketoglutarate (α-KG), producing glutamate (Glu) and the respective branched-chain α-keto acids (BCKAs): α-ketoisocaproate (KIC), α-keto-β-methylvalerate (KMV), and α-ketoisovalerate (KIV). These BCKAs are further decarboxylated by the branched-chain α-keto acid dehydrogenase (BCKDH) complex. Abbreviations: BCAA, branched-chain amino acid; BCAT, branched-chain amino acid transaminase; BCKA, branched-chain α-keto acid; α-KG, α-ketoglutarate; Glu, glutamate; KIC, α-ketoisocaproate; KMV, α-keto-β-methylvalerate; KIV, α-ketoisovalerate; BCKDH, branched-chain α-keto acid dehydrogenase.

The basic metabolic pathways for BCAAs involve transamination, decarboxylation, oxidation, and the formation of final metabolites that enter the tricarboxylic acid cycle (TCA) to provide energy. Among these steps, branched-chain amino acid transaminases (BCATs) are responsible for the transfer of BCAAs, reversibly producing their respective branched-chain α-keto acids (BCKAs), including α-ketoisovalerate (KIC), α-keto-β-methylvalerate (KMV), and α-ketoisovaleric acid (KIV). This process is the first step in the catabolic process of BCAAs. In this step, the amino groupof the BCAA is transferred to α-ketoglutarate (α-KG) to produce glutamate (Toyokawa et al., 2021). A schematic diagram of the BCAA metabolic process is presented in Figure 1.

Two isoforms of BCATs have been identified: BCAT1 (cytoplasmic, BCATc) and BCAT2 (mitochondrial, BCATm). These enzymes catalyse reversible reactions, enabling the transfer of amino groups from glutamate back to BCKAs. This reversibility allows BCATs to regulate the intracellular levels of glutamate and α-KG (Sweatt et al., 2004). Notably, the expression levels of BCAT1 and BCAT2 are closely associated with cancer cell proliferation, invasion, and angiogenesis. Consequently, investigating BCATs has emerged as a rapidly evolving frontier and a significant challenge in oncology. Recent studies have increasingly demonstrated that BCATs play critical roles in tumor formation and drug resistance. Therefore, reviewing the roles and mechanisms of BCATs in various tumor types provides a theoretical foundation and research direction for developing improved cancer treatments and improving patient prognoses.

BCATs are highly expressed in various tumors, including gastric cancer, non-small cell lung cancer, and leukaemia. BCATs can regulate BCAA metabolism through metabolic reprogramming, affecting the levels of metabolic intermediates such as α-KG within cells. This process, in turn, activates survival pathways such as glycolysis through epigenetic modifications (e.g., H3K27 demethylation), providing tumor cells with energy and biosynthetic raw materials (Wang et al., 2019). Additionally, BCAT can regulate the activity of key signalling molecules through acylation or participate in regulating the immune microenvironment (such as influencing the function of tumor-infiltrating immune cells), ultimately promoting tumor cell proliferation, invasion, and distant metastasis. From a clinical perspective, BCATs have been confirmed as potential biomarkers for certain tumors. For example, the overexpression of BCAT1 is positively correlated with the proliferation rate and migration of triple-negative breast cancer (TNBC) cells, and its expression level can predict tumor sensitivity to targeted therapy (Shafei et al., 2020). Therapeutic strategies targeting BCATs are feasible. Additionally, BCAA-restricted diets are garnering attention as an adjunct therapeutic approach. Since BCAT function depends on BCAA metabolism, restricting BCAA intake in the diet may reduce the nutrient supply to tumor cells, synergizing with BCAT-targeted therapy. Therefore, targeting BCATs (including drug inhibition, combination therapy, and BCAA dietary intervention) is a tumor treatment strategy with both a theoretical basis and clinical potential, offering new directions for overcoming tumor resistance and improving patient outcomes.

### 1.1 Background of BCAT

Structurally, BCATs are part of the class IV folded PLP enzymes (Hutson, 2001). BCAT functions as a homodimer. In BCAT, each subunit is composed of a small N-terminus and a large C-terminus connected by an 11-amino acid interdomain loop (Bezsudnova et al., 2020). Both domains adopt an α/β fold. Specifically, the small domain forms an open α/β structure, whereas the large domain forms a pseudobarrel structure. A prominent cavity is formed between the small and large domains of one subunit. Additionally, two loops from the small domain of the adjacent subunit are positioned near this cavity. BCAT enzymes are PLP-dependent homodimers with a unique active site architecture at the subunit interface (Goto et al., 2005). Each active site contains two specialized pockets: an O-pocket that binds hydrophobic BCAA side chains and a P-pocket that specifically anchors α-COOH groups in the substrate (Bezsudnova et al., 2020). The PLP cofactor forms a Schiff base with Lys202, adopting a distinctive orientation that facilitates proton transfer from its reface. This spatial arrangement, where α-carboxyl groups exclusively interact with the P-pocket while side chains occupy the O-pocket, represents a unique feature among transaminases (Bezsudnova et al., 2017).

The human BCATm gene (*BCAT2*), located on chromosome 19, is expressed in most tissues, except the liver. In contrast, the human BCATc gene (*BCAT1*) is located on chromosome 12 and is expressed in very few tissues, such as the brain, ovary, and placental tissue (Hutson et al., 1995; Hutson S, 2001). Mature BCATm (BCAT2), which consists of 365 amino acids, has a molecular weight of approximately 41,300 Da; BCATc (BCAT1) consists of 385 amino acids and weighs approximately 42,800 Da (Davoodi et al., 1998). A monomer of BCATm has smaller structural domains (residues 1–175) and loops that join those domains, plus larger structural domains (residues 176–365). Two PLP cofactors are located at the dimer interface. BCATc consists of a small structural domain (residues 1–188) and a large structural domain with a C-terminus (residues 202–C-terminus), with an interstructural domain loop linked to both (residues 189–201) (Conway et al., 2003). The human BCAT isozymes have 58% amino acid sequence identity. The key active site residues involved in substrate and cofactor binding are nearly identical in the BCATc and BCATm structures (Conway, 2021). This similarity is much more likely to be due to initial amino acid sequences that vary next to the active site. The primary sequence shows that BCATm contains six cysteines with no disulfide bonds. BCATc has 10 cysteines with at least two disulfide bonds. The observed increase in molecular mobility under nonreducing conditions implies that disulfide bond formation contributes to maintaining the structural compactness of the enzyme (Davoodi et al., 1998; Conway, 2021). The catalytic activity of this isozyme is regulated by the redox status of its conserved CXXC motif, which is positioned adjacent to the phosphoryl group of the PLP cofactor. Notably, the redox sensitivity of BCATm is particularly strong, with its structural integrity and functional capacity being significantly influenced by the oxidation state of this motif. Furthermore, this redox-sensitive centre serves as a regulatory switch that can influence intracellular signalling pathways, thereby modulating critical cellular processes, including proliferation and apoptosis (Conway et al., 2004; Herbert et al., 2020). The N-terminal cysteine residues function as redox-sensitive regulatory elements, whereas the C-terminal residues represent solvent-exposed cysteine residues that facilitate reversible enzymatic control (Conway et al., 2002). Although they have the same substrate specificity and almost the same active site, their subtle kinetic variations and distinct responses to redox perturbations indicate potential differential responsiveness to cellular signalling cues.

## 2 BCAT has versatile functions in cancer

### 2.1 BCAT and gastric cancer

Gastric cancer (GC), a leading cause of cancer-related mortality worldwide, has a poor prognosis, despite advances in diagnosis and therapy, and BCAT1/2 dysregulation is closely linked to its progression. Studies have shown that BCAT1 expression is significantly upregulated in GC tissues and that its expression is positively correlated with aggressive clinicopathological features. Importantly, high BCAT1 expression is associated with reduced survival, highlighting its potential as a prognostic biomarker (Xu et al., 2018; Shu et al., 2021). Mechanistically, BCAT1 drives GC progression by activating the phosphatidylinositol 3-kinase (PI3K)/AKT/mammalian target of rapamycin (mTOR) pathway, which is a central regulator of tumor metabolism, proliferation, and metastasis. Preclinical studies have demonstrated that PI3K inhibition reverses BCAT1-driven tumor growth, suggesting that patients with BCAT1-overexpressing GC may benefit from PI3K/AKT/mTOR-targeted therapies. Additionally, BCAT1 enhances angiogenesis *via* vascular endothelial growth factor (VEGF) signalling, upregulating the expression of the markers CD31 and CD34. These findings suggest that BCAT1 is a dual therapeutic target for both direct tumor suppression and antiangiogenic strategies (Shu et al., 2021). Clinically, mTOR inhibitors, such as everolimus and tesilomox, are already approved for use in various cancers, supporting their potential repurposing for the treatment of BCAT1-high GC (Polivka and Janku, 2014). Wang et al. (2020) found that the long noncoding RNA LINC00324 positively modulates BCAT1 expression by binding to microRNA-3200-5p. Silencing LINC00324 or inhibiting miR-3200-5p can alter BCAT1 levels, suggesting that the LINC00324/miR-3200-5p/BCAT1 axis is a potential target for gastric cancer treatment.

**FIGURE 2:**
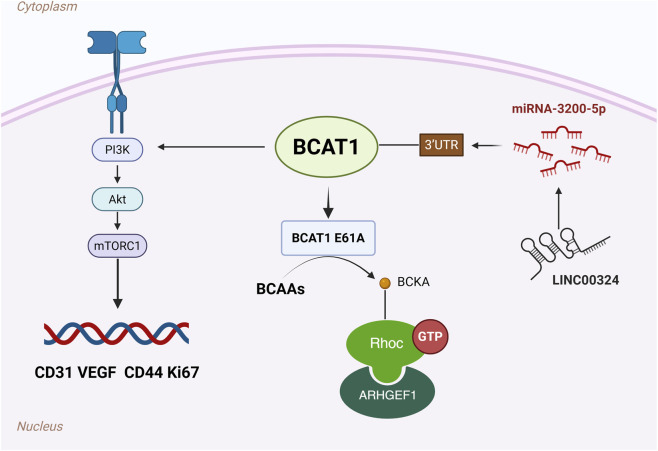
Schematic diagram of the potential mechanism of BCAT1 in gastric cancer (GC). BCAT1 promotes GC progression through multiple mechanisms: (1) Activation of the PI3K/AKT/mTOR signaling pathway and enhancement of VEGF-mediated angiogenesis; (2) Regulation by the LINC00324/miR-3200-5p axis; (3) The E61A gain-of-function mutation enhances BCKA production, which activates RhoC to drive metastasis. Abbreviations: GC, gastric cancer; BCAT1, branched-chain amino acid transaminase 1; PI3K, phosphatidylinositol 3-kinase; mTOR, mammalian target of rapamycin; VEGF, vascular endothelial growth factor; lncRNA, long non-coding RNA; miR, microRNA; BCKA, branched-chain α-keto acid; RhoC, Ras homolog gene family member C.

Qian et al. (2023) identified the BCAT1 E61A mutation in clinical gastric cancer samples and showed that it increases enzymatic activity, increases cellular metabolic activity and migration, and promotes tumorigenesis and progression in multiple organs. The BCAT1 E61A mutation increases BCKA accumulation *via* BCAA catabolism; BCKA binds to and activates the Ras homologue gene family member C (RhoC) to form the BCKA–RhoC–ARHGEF1 complex, indicating that the development of inhibitors is a therapeutic option to block RhoC. Candesartan, a BCAT1 inhibitor, attenuated RhoC activity and cell migration. A low-BCAA diet was also shown to reduce tumor growth in BCAT1 E61A mice. Thus, the inhibition of BCAA metabolism by the diet or drugs could be a potential therapeutic direction. A schematic diagram of the potential mechanism of BCAT1 in GC is shown in Figure 2.

Recent studies have shown that accumulation of leucine in GC activate the mTOR pathway by downregulating BCAT2 expression (Wolfson et al., 2016). Zhang Y. et al. (2022) reported that the expression of BCAT2 is decreased in gastric cancer tissues and that BCAT2 overexpression inhibits the growth of GC cells; thus, BCAT2 may be a candidate target in gastric cancer. Zhang et al. also found that BCAT2 can influence cancer cell survival by affecting mitochondrial metabolism. Moreover, its expression positively correlates with programmed death-1 (PD-1)/cytotoxic T lymphocyte-associated antigen 4 (CTLA-4) expression, suggesting that targeting tumor BCAA metabolism may potentiate immunotherapy efficacy.

In summary, BCAT1 acts as an oncogenic driver in GC through multiple pathways, including PI3K/AKT/mTOR activation, the promotion of angiogenesis, and mutation-mediated RhoC signalling, whereas BCAT2 exerts tumor-suppressive effects, indicating isoform-specific roles that may guide the development of targeted therapy.

### 2.2 BCAT and pancreatic ductal adenocarcinoma

Pancreatic ductal adenocarcinoma (PDAC) relies heavily on BCAA metabolism for progression, with BCAT1 and BCAT2 playing nonredundant roles in tumor cells and the tumor microenvironment, respectively. Compared with that in other organs, the enzymatic activity of BCAT2 is markedly increased in pancreatic exocrine tissue. PDAC cells display increased BCAA uptake and BCAT2 expression, and PDAC cells may utilize BCAAs as a carbon source for fatty acid biosynthesis (Lee et al., 2019; Li et al., 2020). Therefore, BCAT2 plays an indispensable role in supporting PDAC progression. Notably, the suppression of BCAT2 expression results in decreased glutamate output, consequently impairing both glutathione (GSH) biosynthesis and *de novo* nucleotide synthesis. This metabolic disruption increases cellular vulnerability to oxidative damage while simultaneously restraining neoplastic proliferation and expansion (Dey et al., 2017).

#### 2.2.1 Posttranslational regulation of BCAT2 in PDAC

Multiple studies have shown that either genetic knockdown of BCAT2 or pharmacological inhibition combined with a BCAA-restricted diet significantly suppresses pancreatic intraepithelial neoplasia (PanIN) development, reduces PDAC cell proliferation, and prolongs the survival of murine models (Li et al., 2020; Lei et al., 2020; Zhu et al., 2020; Li et al., 2022). The stability and activity of BCAT2 are predominantly governed by posttranslational modifications, which represent a primary layer of regulation in PDAC. Ubiquitin-specific protease 1 (USP1) is an isoenzyme of the BCAT2 deubiquitinase. In both PanIN and PDAC cells, USP1 and BCAT2 are coordinately upregulated and are positively correlated (Li et al., 2022). BCAAs drive USP1 synthesis *via* the general control nonderepressible two–eukaryotic translation initiation factor 2α (GCN2–eIF2α) signalling pathway. Elevated USP1 levels stabilize the BCAT2 protein through deubiquitination at the K229 residue, thereby increasing BCAA utilization and facilitating the progression from PanIN to PDAC. USP1 inhibitors can potentiate BCAT2 ubiquitination and impede PDAC development, suggesting that disruption of BCAT2-mediated BCAA catabolism may represent a viable therapeutic strategy for PDAC. Li et al. (2020) reported that the Kirsten rat sarcoma viral oncogene homologue (KRAS) stabilizes BCAT2 through a dual mechanism involving spleen tyrosine kinase (SYK) and the E3 ubiquitin ligase tripartite motif containing 21 (TRIM21). By inhibiting SYK activity, KRAS reduces BCAT2 phosphorylation at Y228, thereby decreasing TRIM21-mediated ubiquitination and subsequent proteasomal degradation. This KRAS–SYK–TRIM21 axis increases intracellular BCAT2 stability, facilitating PDAC development. BCAT2 protein stability is regulated by acetylation at K44, which promotes ubiquitination-dependent degradation without affecting enzymatic activity (Lei et al., 2020). The K44R mutation stabilizes BCAT2, enhancing BCAA catabolism and pancreatic tumor growth. CREB-binding protein (CBP, acetyltransferase) and sirtuin-4 (SIRT4, deacetylase) modulate BCAT2 acetylation, representing therapeutic targets, while BCAT2 acetylation status may serve as a diagnostic/prognostic biomarker for PDAC.

#### 2.2.2 Transcriptional and metabolic reprogramming in PDAC cells

In addition to posttranslational control, BCAT2 is also subject to transcriptional regulation driven by metabolic stress and genetic alterations. PDAC genomes commonly harbour deletions in tumor suppressor genes such as Sma and Mad-related family member 4 (SMAD4), and deletions of both SMAD4 and malic enzyme 2 (ME2) are common (Dey et al., 2017). ME2 and ME3 are key enzymes involved in mitochondrial malate metabolism and the maintenance of cellular redox homeostasis and energy metabolism. ME2-deficient PDAC cells can upregulate ME3 expression to partially compensate for ME2 loss. However, ME2 deficiency combined with ME3 depletion downregulates BCAT2 expression. Nicotinamide adenine dinucleotide phosphate (NADPH) production is reduced, elevated reactive oxygen species (ROS) levels activate AMP-activated protein kinase (AMPK), and activated AMPK inhibits the nuclear translocation of sterol regulatory element-binding protein 1 (SREBP1) through phosphorylation, which in turn downregulates BCAT2 transcription. The overexpression of BCAT2 results in aggressive tumor growth, confirming the acute dependence of PDAC on BCAAs. This metabolic reprogramming makes PDAC cells more likely to die when they encounter some type of metabolic stress; thus, treatments targeting metabolic reprogramming may be beneficial for people with ME2-deficient PDAC.

#### 2.2.3 Tumor–stromal crosstalk and epigenetic regulation

**FIGURE 3:**
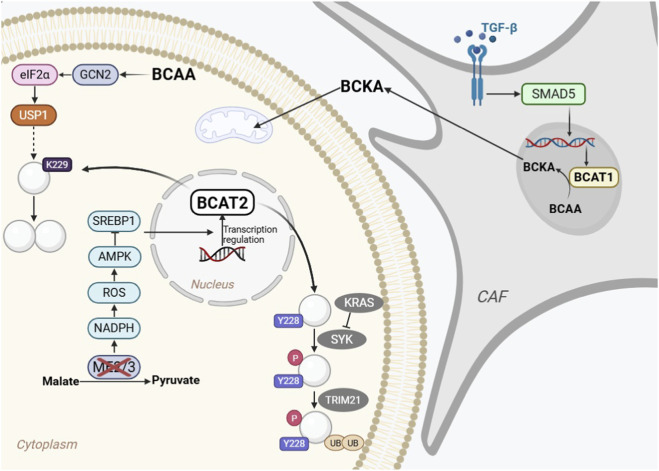
Schematic diagram of the potential mechanism of BCAT1/2 in Pancreatic ductal adenocarcinoma (PDAC). In PDAC cells, BCAT2 is stabilized by various mechanisms (e.g., USP1-mediated deubiquitination, KRAS-SYK-TRIM21 axis, acetylation/deacetylation) to fuel BCAA catabolism. In cancer-associated fibroblasts (CAFs), the TGF-β/SMAD5 axis upregulates BCAT1, which produces BCKAs. These BCKAs are secreted by CAFs and utilized by PDAC cells in a metabolic symbiosis model. Abbreviations: PDAC, pancreatic ductal adenocarcinoma; BCAT1/2, branched-chain amino acid transaminase 1/2; USP1, ubiquitin specific protease 1; KRAS, Kirsten rat sarcoma viral oncogene homolog; SYK, spleen tyrosine kinase; TRIM21, tripartite motif containing 21; TGF-β, transforming growth factor beta; CAF, cancer-associated fibroblast; BCKA, branched-chain α-keto acid.

The regulatory network extends beyond cancer cells to involve complex interactions within the tumor microenvironment. Cancer-associated fibroblasts (CAFs) are crucial cells in the tumor microenvironment. They interact with cancer cells and promote tumor growth and progression by weakening the body’s defence functions. Zhu et al. (2020) reported that under nutrient stress conditions, CAFs maintain the activity of BCAT1 and secrete BCKAs through the internalization of extracellular matrix (ECM) proteins; in contrast, PDAC cells rely on BCAT2-mediated utilization of BCKAs for oxidative metabolism and protein synthesis. Due to their advantages in BCAA metabolism, CAFs create a unique nutritional stress environment for pancreatic ductal adenocarcinoma. BCAT1 is a direct target of the transforming growth factor-β (TGF-β)–SMAD5 pathway in CAFs. TGF-β binds to TGF-β receptors on the surface of CAFs, activating SMAD5, which then binds to the BCAT1 promoter to promote the transcription and expression of BCAT1. The disruption of SMAD5 or BCAA metabolism can inhibit PDAC growth. These findings indicate that the TGF-β/SMAD5 axis in CAFs can directly regulate the expression of BCAT1 and provide amino acid precursors for CAFs to secrete BCKAs through the internalization of the ECM in the tumor microenvironment. A schematic diagram of the potential mechanism of BCAT1/2 in PDAC is shown in Figure 3.

At the epigenetic level, BCAT1 expression is also influenced by RNA modifications. N6-methyladenosine (m6A) RNA methylation is crucial for tumorigenesis and metastasis. A study investigating the role of m6A RNA methylation-related genes in pancreatic cancer identified genes such as BCAT1. These findings might help researchers identify new therapeutic targets and develop potential therapeutic strategies for pancreatic cancer, although the underlying mechanisms still require further investigation (Geng et al., 2020).

In summary, BCAT2 is regulated by multiple posttranslational modifications (ubiquitination and acetylation) and transcriptional mechanisms in PDAC cells, whereas BCAT1 mediates metabolic symbiosis in CAFs, collectively supporting tumor growth; targeting these isoforms or their regulatory pathways may provide synergistic therapeutic benefits for PDAC.

### 2.3 BCAT and non-small cell lung cancer

Non-small cell lung cancer (NSCLC) exhibits distinct BCAT1 expression patterns and functional roles, with epigenetic regulation and crosstalk between pathways driving tumor progression and drug resistance. An analysis of The Cancer Genome Atlas (TCGA) data and an independent NSCLC cohort revealed extensive BCAT1 hypermethylation in NSCLC, accompanied by the significant downregulation of BCAT1 expression in tumor tissues, indicating the direct epigenetic regulation of this gene (Diaz-Lagares et al., 2016). Symonds et al. analysed methylated BCAT1/IKAROS family zinc finger 1 (IKZF1) circulating tumor DNA (ctDNA) and showed that it is highly sensitive for detecting lung adenocarcinoma and has potential clinical utility as an adjunct diagnostic method for assessing malignancy (Mohamed et al., 2024). Notably, increasing ctDNA levels correlated with disease progression, suggesting that this assay may be particularly valuable for detecting advanced-stage tumors. Multiple studies have reported elevated BCAT1 protein expression in NSCLC tissue compared with normal lung tissue (Lin et al., 2020; Yu et al., 2022). This overexpression is positively associated with lymph node metastasis, an advanced TNM stage, and a poor prognosis, which increases NSCLC cell proliferation, migration, and invasion, supporting its role in tumor aggressiveness. Although DNA methylation represents a significant regulatory level, it is not the sole mechanism. This apparent contradiction between mRNA downregulation and protein overexpression may be explained by posttranscriptional regulation or posttranslational modifications that prevent protein degradation, highlighting the complexity of the regulatory mechanisms of BCAT1 in NSCLC.

Nuclear transcription factor-κB (NF-κB) is related to apoptosis, tumorigenesis, inflammation, and metabolism. Typically, elevated antioxidant levels and low ROS concentrations activate NF-κB signalling, which further impairs ROS production and antioxidant expression (Morgan and Liu, 2011). Research conducted by Yu et al. (2022) revealed that lung adenocarcinoma (LUAD) cells overexpressing BCAT1 display decreased ROS accumulation and elevated antioxidant expression, potentially activating the NF-κB pathway. These observations strongly suggest that BCAT1-mediated NF-κB activation significantly contributes to LUAD progression. Lin et al. (2020) showed that BCAT1 overexpression also stimulates the Wnt/β-catenin signalling cascade. An analysis of TCGA datasets confirmed the positive regulatory effect of BCAT1 on key Wnt pathway targets, including the oncogenes myelocytomatosis viral oncogene homologue (c-Myc), cyclin D1, and matrix metalloproteinase 7 (MMP7). Further experiments showed that BCAT1 upregulates the active β-catenin protein while downregulating its phosphorylated, inactive form. When Wnt signalling was pharmacologically inhibited, the effects of BCAT1 on the expression of c-Myc, cyclin D1, and MMP7 were substantially decreased, with a concurrent modest reduction in BCAT1 levels. These findings collectively establish a novel BCAT1–Wnt/β-catenin regulatory feedback loop involved in LUAD pathogenesis.

A study revealed that compared with primary tumor cells, metastatic lung cancer cells exhibit significantly higher BCAT1 expression (Mao et al., 2021). Genetic silencing of BCAT1 not only attenuated cancer cell migration *in vitro* but also markedly decreased the bone metastasis burden *in vivo*, suggesting that BCAT1 overexpression may functionally drive lung cancer metastasis. Their study further revealed a mechanistic link between BCAT1 and the stemness regulator SRY-box transcription factor 2 (SOX2). Elevated BCAT1 levels can promote SOX2 expression through α-KG deficiency, with α-KG serving as a crucial cofactor for DNA demethylase 10–11 translocation 2 (TET2). This reduction in α-KG expression results in hypermethylation of target gene promoters, leading to their transcriptional silencing, and impairs TET activity, causing decreased miR-200c expression. Notably, supplementation with exogenous α-KG reversed these effects, reducing SOX2 levels while restoring miR-200c expression in metastatic cells. These findings reveal a novel BCAT1/α-KG/miR-200c/SOX2 regulatory axis in metastatic lung cancer and suggest potential therapeutic approaches for targeting this pathway.

**FIGURE 4:**
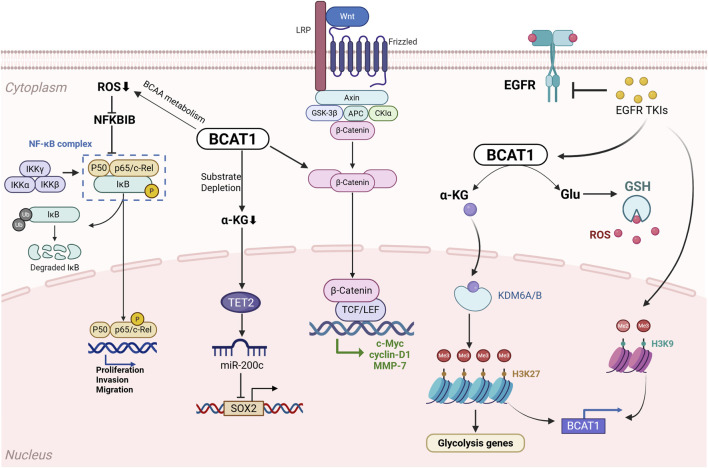
Schematic diagram of the potential mechanism of BCAT1 in non-small cell lung cancer (NSCLC). BCAT1 drives NSCLC progression *via*: (1) Reducing reactive oxygen species (ROS) and activating NF-κB signaling; (2) Activating the Wnt/β-catenin pathway; (3) The BCAT1/α-KG/miR-200c/SOX2 axis promoting metastasis and stemness; (4) Conferring resistance to EGFR tyrosine kinase inhibitors (TKIs) through epigenetic activation of glycolysis and suppression of ROS. Abbreviations: NSCLC, non-small cell lung cancer; BCAT1, branched-chain amino acid transaminase 1; ROS, reactive oxygen species; NF-κB, nuclear factor kappa-light-chain-enhancer of activated B cells; α-KG, α-ketoglutarate; miR, microRNA; SOX2, SRY-box transcription factor 2; EGFR, epidermal growth factor receptor; TKI, tyrosine kinase inhibitor.

The novel BCAT1 inhibitor WQQ-345 has been shown to exhibit significant antitumor activity in both *in vitro* and *in vivo* models of tyrosine kinase inhibitor (TKI)-resistant lung cancer (Zhang et al., 2024). Glutamate, a key metabolic product of BCAT1 activity, serves as a crucial precursor for GSH biosynthesis. As a potent antioxidant, GSH plays a critical role in scavenging reactive ROS and protecting cells from oxidative damage. Interestingly, epidermal growth factor receptor (EGFR) TKI treatment induces ROS accumulation in cancer cells, and recent research has revealed that BCAT1 protects against EGFR TKI toxicity by increasing GSH production, thereby enhancing cellular antioxidant defences and reducing ROS levels (Wang et al., 2019). Persistent exposure to sublethal doses of TKIs can induce transient drug tolerance in EGFR-mutant lung cancer cells, ultimately leading to acquired TKI resistance. Mechanistic studies have demonstrated that in these sublethal TKI-adapted cells (STACs), BCAT1 expression is upregulated through histone H3 lysine 9 (H3K9) demethylation-mediated epigenetic activation. A chromatin immunoprecipitation analysis confirmed decreased H3K9me2 and H3K9me3 levels at the BCAT1 promoter region in STACs. Genetic knockdown of BCAT1 resulted in markedly increased intracellular ROS levels, restored EGFR TKI sensitivity, and suppressed tumor growth. These findings suggest that combining EGFR TKIs with ROS-inducing agents may represent an effective strategy to overcome drug resistance in STACs. Collectively, these findings indicate that BCAT1 promotes resistance to third-generation EGFR TKIs through two key mechanisms: epigenetic activation of glycolytic pathways and the suppression of ROS accumulation in NSCLC. These insights strongly support BCAT1 as a promising therapeutic target for managing TKI-resistant NSCLC. A schematic diagram of the potential mechanism of BCAT1 in NSCLC is shown in Figure 4.

### 2.4 BCAT and leukaemia

Leukaemia progression is characterized by BCAT1-mediated metabolic reprogramming and epigenetic dysregulation, with isoform-specific effects on different leukaemia subtypes. In normal haematopoiesis, enhancer of zeste homologue 2 (EZH2)-mediated H3K27 trimethylation silences BCAT1 expression. This repression is lost in EZH2-deficient myeloid malignancies, leading to aberrant BCAT1 activation that cooperates with neuroblastoma NRAS G12D mutations to accelerate the progression from myeloproliferative neoplasms (MPNs) to aggressive leukaemia (Gu et al., 2019). Mechanistically, BCAT1 maintains intracellular BCAA pools through reversible reactions, sustaining mTOR pathway hyperactivation to drive leukaemic transformation. Notably, BCAT1 deletion has no effect on normal haematopoietic stem or progenitor cells; however, BCAT1 inhibition can selectively target EZH2-deficient leukaemia cells, opening up a therapeutic window. The combination of BCAT1 inhibitors with mTOR inhibitors or restricting dietary BCAAs could serve as potential therapeutic strategies. This study reveals how epigenetic dysregulation reprograms metabolic networks during leukaemogenesis and confirms that BCAT1 is a clinically actionable target.

Emerging evidence has indicated that BCAT1 modulates DNA damage repair mechanisms in acute myeloid leukaemia (AML) through the regulation of α-KG levels (Raffel et al., 2017; Pan et al., 2024). Pan et al. (2024) revealed that high expression of BCAT1 inhibits α-KG-dependent histone demethylase activity and impairs KDM4A/B activity by reducing α-KG levels, thereby upregulating H3K9me3 levels. This epigenetic alteration suppresses ataxia telangiectasia-mutated gene (ATM) expression and impairs DNA repair, increasing the vulnerability of AML cells to DNA-damaging agents. Notably, BCAT1-overexpressing AML cells show increased sensitivity to the poly (ADP-ribose polymerase) (PARP) inhibitor BMN673. These findings suggest that BCAT1 is both a prognostic biomarker and a predictor of the therapeutic response, suggesting that BMN673 is a promising treatment for patients with BCAT1-high AML. The metabolic regulator α-KG serves as an essential cofactor for multiple enzyme families, such as the Egl-9 family of hypoxia-inducible factors (Egl nine homologue 1, EGLN1) and the TET family of DNA demethylation enzymes. Raffel et al. (2017) found that BCAT1 knockdown increases α-KG levels, activating EGLN1 to degrade hypoxia-inducible factor-1 (HIF-1α) and impair leukaemia stem cell survival. Conversely, BCAT1 overexpression reduces α-KG levels, inhibits TET2 and stabilizes HIF-1α to result in a hypermethylated phenotype similar to that of the IDH-mutant type. Clinically, high BCAT1 levels predict a poor prognosis, especially in patients with isocitrate dehydrogenase (IDH)/TET2 wild-type (WT) AML, suggesting that the BCAT1–α-KG axis is a promising therapeutic target for this subset. METTL16, an m6A methyltransferase overexpressed in AML and leukaemia stem cells (LSCs), strongly binds to multiple m6A-modified sites within the BCAT1/2 mRNAs (Han et al., 2023). Through this interaction, METTL16 promotes AML by stabilizing BCAT1/2 mRNAs *via* m6A-YTH domain-containing protein 1 (YTHDC1) signalling. This metabolic reprogramming increases BCAA metabolism, driving LSC self-renewal and AML progression while sparing normal haematopoiesis, highlighting METTL16 as a promising therapeutic target.

Shao et al. (2024) reported that BCAT1 is aberrantly expressed in 30% of chronic lymphocytic leukaemia (CLL) patients, particularly those with del17p/TP53 mutations or trisomy 12. In CLL cells, BCAT1/2 exhibits bidirectional responses, which promote mTOR activation *via* leucine/acetyl-CoA/α-KG production, driving CLL progression. CLL patients with aberrant BCAT1 expression had shorter survival times than those with BCAT1-negative CLL, potentially because of enhanced BCR/mTOR signalling. In CLL, BCAA utilization in the TCA cycle is mediated primarily by mitochondrial BCAT2. BCAT2 deficiency upregulates BCAT1, revealing metabolic compensation. Targeting BCAT1/mTOR may benefit high-risk CLL patients.

**FIGURE 5:**
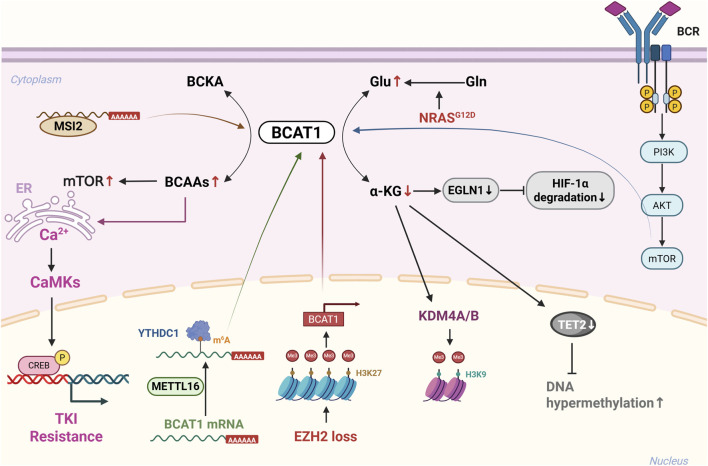
Schematic diagram of the potential mechanism of BCAT1 in leukemia. BCAT1 is aberrantly activated in various leukemias: (1) Loss of EZH2-mediated repression in myeloid malignancies; (2) Regulation by MSI2 in blast crisis chronic myeloid leukemia (BC-CML); (3) Modulation of α-KG levels, affecting epigenetic regulators (KDM4, TET2) and DNA damage repair; (4) METTL16-mediated m6A methylation stabilizes BCAT1/2 mRNA; (5) Activation of mTOR signaling and calcium-CREB pathway. Abbreviations: BCAT1, branched-chain amino acid transaminase 1; EZH2, enhancer of zeste homolog 2; MSI2, Musashi RNA-binding protein 2; BC-CML, blast crisis chronic myeloid leukemia; α-KG, α-ketoglutarate; KDM4, lysine demethylase 4; TET2, ten-eleven translocation 2; METTL16, methyltransferase-like 16; m6A, N6-methyladenosine; mTOR, mammalian target of rapamycin; CREB, cAMP response element-binding protein.

Hattori et al. (2017) identified a Musashi RNA-binding protein 2 (MSI2)–BCAT1 regulatory axis that drives blast crisis chronic myelogenous leukaemia (BC-CML) progression. MSI2 binds a specific sequence in the 3′-UTR of the BCAT1 mRNA, increasing its expression and enabling BCAT1 to reverse its enzymatic activity to regenerate BCAAs, sustaining mTORC1 signalling *via* S6K phosphorylation (pS6K). MSI2 knockdown reduces BCAT1 levels, impairing mTOR activation and leukaemia growth. This axis represents a therapeutic vulnerability in myeloid malignancies. BCAT1 plays oncogenic roles in multiple leukaemia subtypes through α-KG-mediated epigenetic regulation, the modulation of DNA damage repair, and activation of the mTOR pathway, whereas BCAT2 is involved in metabolic compensation in CLL, indicating its potential for subtype-specific targeting. A schematic diagram of the potential mechanism of BCAT1 in leukaemia is shown in Figure 5.

### 2.5 BCAT and gliomas

Gliomas, the most aggressive primary intracranial tumors, exhibit distinct metabolic features driven by the IDH mutation status, and BCAT1 has emerged as a key oncogenic mediator, particularly in IDH1-wild-type (IDH1-WT) subtypes. Gliomas are common primary brain tumors, and the mutation status of isocitrate dehydrogenase 1 (IDH1) is important for the classification of gliomas. Based on the IDH mutation status, gliomas can be classified as primary (IDH1/2-WT) or secondary (IDH1/2-mut). BCAT1 can be used as a biomarker associated with magnetic resonance imaging of gliomas (Chaumeil et al., 2014). BCAT1 catalyses the transamination of BCAA while converting α-KG to glutamate. A study using 13C magnetic resonance spectroscopy with hyperpolarized [1-(13) C] α-KG as a probe revealed that the level of hyperpolarized [1-(13) C] glutamate was significantly reduced in IDH1-mut glioma cells and tumors and could be used as a metabolic imaging biomarker for the IDH1 mutation status. Another study (Cho et al., 2017) showed that BCAT1 could be used as a biomarker for IDH1-WT glioblastoma (GBM); the overexpression of BCAT1 is associated with high cerebral blood volume (CBV) and a low apparent diffusion coefficient (ADC) and a poor prognosis, and BCAT1 activity has also been assessed using imaging.

In IDH-WT tumors, BCAT1 expression is much higher than that in both IDH-mut tumors and normal tissue; thus, BCAT1 expression could help distinguish between primary and secondary gliomas. Elevated expression has been detected in GBM and relatively low-grade gliomas and is correlated with high levels of DNA methylation and epigenetic silencing of the BCAT1 promoter region (Tönjes et al., 2013). McBrayer et al. (2018) found that (R)-2-hydroxyglutarate (2HG) produced by IDH1/2 mutants inhibits the activity of BCATs in a variety of ways, including direct competition for substrates, influencing enzyme structure and function, and metabolic regulation, leading to impaired BCAA metabolism and affecting glutamate biosynthesis in glioma cells, which in turn decrease glutathione levels and cause an imbalance in redox homeostasis. Under hypoxic conditions, HIF-1 can upregulate BCAT1 mRNA and protein expression levels in glioblastoma cells by binding to the hypoxia response element of the BCAT1 gene (Zhang et al., 2021a). Metabolic tracer experiments showed that hypoxia increased nitrogen transfer from BCAAs to glutamate, which was eliminated by HIF-1α knockdown, suggesting that HIF is a key regulator of BCAA metabolic reprogramming in glioblastoma cells in response to hypoxia.

The expression of BCAT1 also varies widely among glioblastomas, and the effect of BCAT1 on glioblastoma proliferation and invasion is also subtype specific (Fala et al., 2023). In glioblastomas with elevated BCAT1 expression, BCAT1 overexpression promotes proliferation and invasion by downregulating α-KG, reducing TET activity to increase DNA hypermethylation, and increasing the stability of HIF-1α to upregulate Forkhead box protein M1 (FOXM1) while maintaining a cancer stem-like cell state. However, it reduces proliferation in cancer cells with low BCAT1 expression. IDH-WT glioma cells depend on BCAA metabolism to sustain their growth. Knockdown or inhibition of BCAT1 leads to BCAA accumulation and reduced glutamate release, thereby inhibiting cell proliferation, inducing G1 phase arrest, and slowing tumor growth. BCAT1 actively participates in multiple oncogenic pathways in IDH1-WT gliomas, including cell death regulation, the oxygen deprivation response, angiogenesis, glycolytic metabolism, and immunosuppression. It also has significant potential as both a diagnostic indicator and a prognostic evaluation tool for glioma patients, with elevated expression levels serving as a reliable predictor of unfavourable clinical outcomes for IDH1-WT patients.

BCAT1 plays a key role in maintaining the undifferentiated state of GBM cells and regulating cellular plasticity and the immunosuppressive microenvironment (Boskovic et al., 2024). BCAT1 serves as a key regulator of cellular plasticity in GBM, where it maintains undifferentiated cell states through DNA methylation-mediated inhibition of cellular differentiation while simultaneously promoting immunosuppression *via* the modulation of the tumor immune microenvironment. BCAT1 knockout cells exhibited distinct differentiation features, such as an altered cell morphology and increased expression of neural differentiation molecules, as well as remodelling of the tumor immune microenvironment and increased CD8^+^ T-cell infiltration. These results reveal a novel treatment strategy for GBM through BCAT1 suppression.

Noncoding RNAs regulate glioma progression through BCAT1. The long noncoding RNA (lncRNA) PSMB8-AS1 upregulates BCAT1 expression by competitively binding to miR-382-3p (Liu et al., 2024), whereas circRNA VPS18 functions as a molecular sponge to sequester miR-1229-3p, thereby alleviating its suppression of BCAT1 (Huang et al., 2022). The activation of BCAT1 promotes glioma cell proliferation, invasion, angiogenesis, and apoptosis resistance, establishing BCAT1 as a critical therapeutic target for glioma treatment.

**FIGURE 6:**
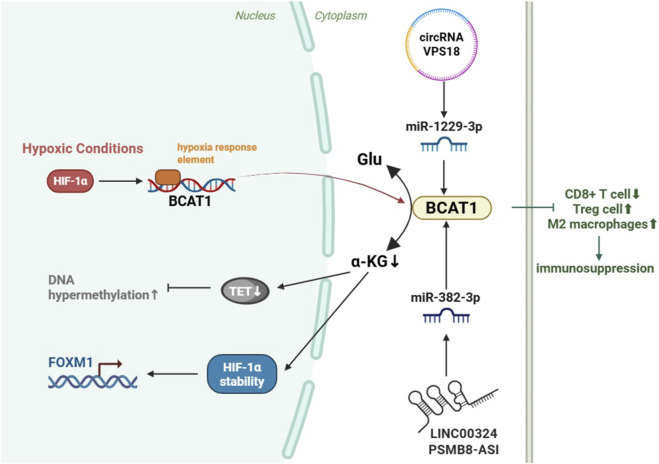
Schematic diagram of the potential mechanism of BCAT1 in glioma. In IDH1-wildtype glioblastoma (GBM), BCAT1 expression is upregulated by HIF-1 under hypoxia and non-coding RNAs (e.g., circVPS18, lncRNA PSMB8-AS1). BCAT1 promotes tumor progression by depleting α-KG, leading to DNA hypermethylation, HIF-1α stabilization, FOXM1 upregulation, and maintenance of a stem-like state. It also regulates cellular plasticity and contributes to an immunosuppressive microenvironment. Abbreviations: IDH1, isocitrate dehydrogenase 1; GBM, glioblastoma; BCAT1, branched-chain amino acid transaminase 1; HIF-1, hypoxia-inducible factor-1; circRNA, circular RNA; lncRNA, long non-coding RNA; α-KG, α-ketoglutarate; FOXM1, forkhead box protein M1.

Zhang B. et al. (2022) reported that targeting BCAT1 in combination with α-KG is a novel anabolic lethal approach for treating IDH WT GBM without significant toxicity to normal mouse astrocytes. BCAT1 inhibition plus α-KG induces synthetic lethality in IDH-WT GBM by increasing the NAD^+^/NADH ratio, disrupting mitochondrial function, suppressing mTORC1 synthesis, and affecting nucleotide generation while sparing normal astrocytes. These changes highlight its clinical potential. BCAT1 is a central oncogenic driver in IDH1-WT gliomas and regulates metabolism and immunosuppression through multiple mechanisms, including the hypoxia response and noncoding RNA crosstalk, with combination therapies targeting BCAT1 showing promising preclinical efficacy. A schematic diagram of the potential mechanism of BCAT1 in glioma is shown in Figure 6.

### 2.6 BCAT and breast cancer

BCAT1 drives breast cancer (BC) progression and therapeutic resistance through metabolic reprogramming and the activation of signalling pathways, with distinct effects on subtypes such as triple-negative breast cancer (TNBC). Studies have shown that both BCAA levels and BCAT1 expression are high in BC tissues (Zhang and Han, 2017). BCAT1 is highly expressed in both human breast cancer cells and mouse models, supporting cell growth and survival. It promotes colony formation in BC cells and regulates mitochondrial biogenesis and function; specifically, it increases the mitochondrial content by upregulating the expression of peroxisome proliferator-activated receptor-gamma coactivator-1α (PGC-1α), nuclear respiratory factor-1 (NRF-1), mitochondrial transcription factor A (TFAM), and beta-subunit F1 ATPase (β-F1-ATPase). Additionally, BCAT1 enhances mitochondrial function by activating mTOR signalling, increasing citrate synthase activity and cytosolic ATP levels, and reducing cytosolic mitochondrial ROS levels.

Disruptor of telomeric silencing 1-like (DOT1L) is essential for the transformation of breast epithelial cells, breast tumorigenesis and metastasis. Its interaction with c-Myc-p300 during the epigenetic activation of epithelial–mesenchymal transition (EMT)-related TFs during breast cancer progression is important (Oktyabri et al., 2016). In a study by Oktyabri et al., BCAT1 was identified as one of the target genes of DOT1L, which regulates BCAT1 expression through histone H3K79 methylation to control the sphere formation and migration of BC cell lines. These findings indicate that BCAT1 expression is epigenetically regulated, providing an upstream mechanism for its overexpression in breast cancer and integrating this regulatory layer into the overall narrative of BCAT1-driven oncogenesis. Targeting BCAT1 reduces the energy supply and metabolic adaptations of tumor cells and may be a new strategy for the treatment of BC.

**FIGURE 7:**
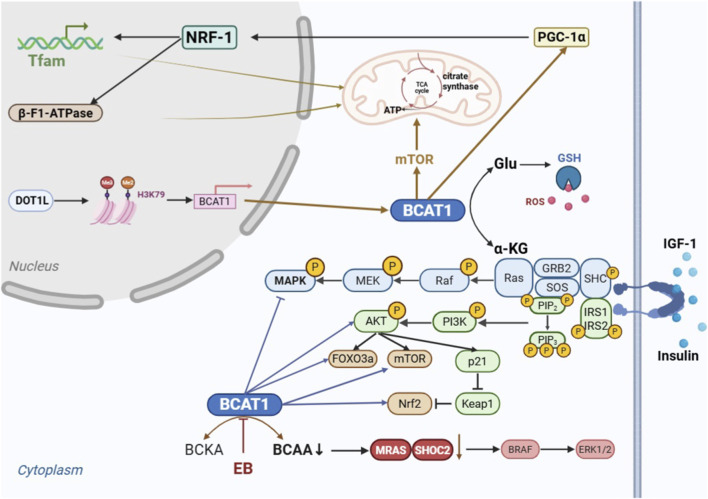
Schematic diagram of the potential mechanism of BCAT1 in breast cancer (BC). BCAT1 promotes BC, particularly triple-negative breast cancer (TNBC), by: (1) Enhancing mitochondrial biogenesis and function *via* the mTOR pathway; (2) Being transcriptionally regulated by DOT1L; (3) Activating the IGF-1/PI3K/Akt, IGF-1/Ras/MAPK and SHOC2-RAS-ERK signaling pathways to drive proliferation and survival. Abbreviations: BC, breast cancer; TNBC, triple-negative breast cancer; BCAT1, branched-chain amino acid transaminase 1; mTOR, mammalian target of rapamycin; DOT1L, disruptor of telomeric silencing 1-like; IGF-1, insulin-like growth factor 1; PI3K, phosphatidylinositol 3-kinase; Akt, Protein Kinase B; Ras, Rat sarcoma viral oncogene homolog; MAPK, Mitogen-activated protein kinase; EB, Eupalinolide B; SHOC2,soc-2 suppressor of clear homolog; MRAS, muscle RAS oncogene homolog; ERK, Extracellular signal-regulated kinase.

Triple-negative breast cancer (TNBC), the most aggressive breast cancer subtype with limited treatment options and poor clinical outcomes, is strongly associated with elevated BCAT1 expression and an unfavourable prognosis; thus, BCAT1 was established as an independent prognostic biomarker (Shafei et al., 2020; Song et al., 2020). BCAT1 drives TNBC progression by activating the IGF-1/PI3K/Akt axis to promote proliferation, migration, and invasion and by modulating the RAS/ERK pathway through the upregulation of Forkhead box O3a (FOXO3a) and nuclear factor erythroid 2-related factor 2 (Nrf2), altering cellular redox homeostasis to support tumor survival. These findings suggest that BCAT1 is a promising therapeutic target in TNBC (Shafei et al., 2020). SHOC2 (soc-2 suppressor of clear homolog) is a leucine-rich repeat-containing scaffold protein that typically forms a complex with MRAS (muscle RAS oncogene homolog), Huang et al. (2025) revealed that Eupalinolide B (EB), a natural small molecule derived from Eupatorium lindleyanum, exerts antitumor effects. Specifically, EB binds to the Cys335 residue of BCAT1, inhibiting its activity to reduce BCAA biosynthesis and suppress SHOC2–RAS–ERK signalling, thereby inducing TNBC cell apoptosis. These findings indicate that BCAT1 promotes oncogenesis by enhancing SHOC2 expression and activating the downstream RAS-ERK oncogenic signaling pathway, thereby providing new therapeutic avenues for EB- or BCAT1-targeted strategies. BCAT1 plays critical roles in breast cancer by regulating mitochondrial function, epigenetic pathways, and oncogenic signalling cascades, with targeted inhibition showing the potential to overcome therapeutic resistance in TNBC and other subtypes. These findings suggest that BCAT1 promotes oncogenesis by enhancing SHOC2 expression and activating the downstream RAS-ERK oncogenic signaling cascade. A schematic diagram of the potential mechanism of BCAT1 in BC is shown in Figure 7.

### 2.7 BCAT and hepatocellular carcinoma

BCAAs have been shown to exert antifibrotic effects by eliminating the negative feedback regulation of mTORC1 on TGF-β1 signalling, preventing hepatocyte apoptosis, and reducing the incidence of hepatocellular carcinoma (HCC) (Takegoshi et al., 2017). In HCC, BCAT1 may affect the energy supply and metabolic status of tumor cells by regulating BCAA metabolism, which in turn affects tumor growth and development.

BCAT1 expression is significantly increased in HCC tissues and cell lines compared with normal control tissues (Xu et al., 2016; Zheng et al., 2016; Zou et al., 2019; Ding et al., 2023). BCAT1 upregulation contributes to HCC pathogenesis by enhancing multiple oncogenic properties, including cellular proliferation, clonogenic potential, tumor migratory behaviour, and invasion. Clinically, elevated BCAT1 expression is strongly correlated with unfavourable outcomes for patients with HCC. Mechanistic studies revealed that BCAT1 expression is positively correlated with the expression of the oncoprotein c-Myc, which directly activates the BCAT1 promoter, thereby establishing a feed-forwards regulatory loop that drives HCC growth and progression (Xu et al., 2016; Zheng et al., 2016). These findings collectively suggest that BCAT1 is both a prognostic biomarker and a potential therapeutic target in HCC. BCAT1 may be involved in increasing HCC cell invasion and migration by promoting AKT activity and inducing the EMT (Ding et al., 2023). Following the silencing of BCAT1 or inhibition of AKT, AKT expression in HCC tissues is positively correlated with BCAT1, which promotes cancer cell proliferation. The downregulation of BCAT1 alters the expression of BCAT1-related factors such as Twist, E-cadherin, and vimentin, thereby preventing the EMT. BCAT1 collectively drives the malignant progression of HCC by coordinating metabolic reprogramming (*via* BCAA catabolism), transcriptional regulation (*via* c-Myc), and the activation of signalling pathways (*via* AKT), making it a multifaceted target for HCC therapy.

Mesenchymal stromal cell (MSC) implantation is promising for the treatment of liver disease and has been applied in clinical settings. However, the poor retention of MSCs in the injured liver environment significantly impairs their therapeutic efficacy. In particular, ferroptosis is the main cause of early MSC loss after implantation in injured livers or after ROS stress, with substantial loss within 6 h of implantation accounting for the majority of the total cellular depletion. Ferroptosis is an iron-dependent mode of cell death driven by cellular metabolism and the iron-dependent accumulation of lipid peroxidation. BCAT1 expression is sensitive to ROS stress and ferroptotic stimuli, and its downregulation renders MSCs susceptible to ferroptosis; moreover, BCAT1 is a key molecule that regulates the resistance of MSCs to ferroptosis under ROS stress (Hu et al., 2023). BCAT1-overexpressing MSCs showed increased resistance to ferroptosis. In ferroptotic or ROS-induced MSCs, BCAT1 expression was significantly reduced, which sensitized MSCs to ferroptosis through a coordinated metabolic–epigenetic mechanism mediated by increased α-KG accumulation, inhibition of the H3K9me3 modification, upregulated EGR1 expression, and the inhibition of glutathione peroxidase-4 (GPX4) transcription by binding to the promoter region. The overexpression of BCAT1 or ferroptosis inhibitors increased the resistance of MSCs to ferroptosis in a GPX4-dependent manner. In addition, the implantation of MSCs overexpressing BCAT1 in the injured liver could reduce hepatic necrotic lesions, protect liver function, reduce inflammatory cell infiltration and proinflammatory cytokine production, and improve therapeutic efficacy.

### 2.8 BCAT and other tumors

BCAT1/2 dysregulation is a common feature of diverse tumor types and plays isoform-specific roles in metabolic reprogramming, therapeutic resistance, and tumor–stromal crosstalk. BCAT1 drives colorectal cancer (CRC) progression through the TMPO-AS1/miR-98-5p/BCAT1 axis, where the long noncoding RNA TMPO-AS1 sponges miR-98-5p to upregulate BCAT1, increasing cell proliferation (Ye et al., 2022). Yoshikawa et al. (2006) identified BCAT1 as a biomarker of metastasis that is inhibited by PSK39, suggesting its therapeutic potential. The methylation of BCAT1 in ctDNA is a highly specific and sensitive diagnostic marker for CRC, outperforming CEA levels and faecal immunochemical tests (FITs) (Symonds et al., 2016; Young et al., 2016). Posttreatment monitoring of methylated BCAT1/IKZF1 in ctDNA may predict recurrence (Pedersen et al., 2023).

Nasopharyngeal carcinoma (NPC) is highly prevalent in southern China and Southeast Asia. Due to the absence of symptoms in its early stages, it is often diagnosed at an advanced stage. BCAT1, an early pathogenic driver, is overexpressed in precancerous lesions through gene amplification and c-Myc-mediated transcriptional regulation (Zhou et al., 2013). A study revealed that the flotillin-2 (FLOT2)/miR-33b-5p/c-Myc axis plays a key role in regulating BCAT1 (Liu et al., 2021). The membrane protein FLOT2 inhibits the expression of miR-33b-5p, alleviating its suppression of c-Myc, and c-Myc can bind to the 5′ promoter region of BCAT1 to increase its expression. Knocking down BCAT1 expression impaired the proliferation, migration, and invasion of NPC cells, and its overexpression was associated with a poor prognosis. This regulatory axis provides a potential target for the intervention and treatment of NPC.

Compared with normal prostate tissue, miR-218 expression is markedly downregulated in prostate cancer (PCa) tissues, where it functions as a tumor suppressor and increases cancer cell sensitivity to cisplatin (CDDP) (Zhu et al., 2017). Bioinformatics analysis and experimental validation revealed that BCAT1 is a direct downstream target of miR-218 and that its overexpression can counteract the tumor-suppressive effects of miR-218 and induce CDDP resistance in PCa cells, establishing BCAT1 as a critical mediator of the therapeutic response. In addition, Src can promote androgen receptor (AR) expression by activating BCAT1 and inducing the proliferation of androgen-independent prostate cancer cells (Chattopadhyay et al., 2017). Therefore, targeting miR-218 or BCAT1 in combination with CDDP therapy may improve the efficacy of PCa treatments. Treatments targeting valine catabolism significantly inhibit the growth of castration-resistant prostate cancer (CRPC). BCAT1 is abnormally highly expressed in enzalutamide-resistant CRPC and is closely associated with valine-driven fatty acid uptake and succinate metabolism, collectively forming the core of metabolic reprogramming in cancer cells. BCAT1 is the key molecule enabling prostate cancer cells to switch from the “leucine dependence of nonmalignant cells” to the “valine dependence of malignant cells”. Furthermore, BCAT1 and BCAT2 exhibit a “transcriptional compensatory relationship,” together maintaining the stability of BCAA catabolism (Bidgood et al., 2024). Studies have shown that BCAT2 can inhibit autophagy, autophagy-dependent apoptosis and ferroptosis in PCa (Mei et al., 2025). As a downstream protein of BCAT2, PCBP1 interacts with BCAT2 at leucine 239, regulating the PI3K/AKT signalling pathway and influencing the progression of PCa.

BCAT1 drives the EMT in KIRC cells by directly downregulating the expression of epithelial markers (e.g., E-cadherin) and upregulating the expression of mesenchymal markers (e.g., vimentin) (Li et al., 2024). In melanoma, BCAT1 induces glycolytic metabolism through c-Myc (Zhang et al., 2021b), whereas BCAT2 regulates FA synthase (FASN) and ATP-citrate lyase (ACLY) through P300-dependent histone acetylation to promote *ab initio* lipogenesis, leading to tumor growth and metastasis, and ZEB1 is identified as the upstream transcriptional activator of BCAT2 (Tian et al., 2023). In endometrial cancer (EC), BCAT1 activates mTORC1 *via* BCAA production (Wang et al., 2018).

In bladder cancer, BCAT2 suppresses cytotoxic T-cell recruitment, creating a noninflammatory tumor microenvironment (TME) (Cai et al., 2023). Its inhibition synergizes with anti-PD-1 therapy, increasing CD8^+^ T-cell infiltration and cytotoxicity. BCAT2 expression is negatively correlated with the immunotherapy response, suggesting its potential as a target for combination immune checkpoint blockade.

Across diverse tumor types, BCAT1 functions primarily as an oncogenic driver that activates metabolic and signalling pathways, whereas BCAT2 plays context-dependent roles (tumor-suppressive or oncogenic) and is involved in metabolic compensation; targeting these isoforms holds promise for personalized therapy across cancer subtypes.

## 3 BCAT is a promising target for tumor treatment

BCATs (BCAT1 and BCAT2) have become key potential targets in tumor therapy, as they play core roles in tumor metabolic reprogramming, malignant growth, and drug resistance. By regulating BCAA metabolism, BCATs supply tumor cells with energy and biosynthetic materials for proliferation. They also drive tumor development and resistance by interacting with oncogenic pathways, affecting epigenetic changes and the immune microenvironment. Preclinical studies have shown that targeting BCATs can inhibit tumor growth, block metastasis, and reverse therapeutic resistance due to metabolic reprogramming. Below, we analyse how BCAT inhibition restores tumor sensitivity to chemotherapy, endocrine therapy, and targeted therapy to highlight the clinical value of BCAT-targeted treatment.

### 3.1 The inhibition of BCAT resensitizes cells to chemotherapy

Tyrosine kinase inhibitors (TKIs) are a class of important targeted anticancer drugs that play key roles in the treatment of various tumors, including non-small cell lung cancer, chronic myeloid leukaemia, gastrointestinal stromal tumors, and renal cell carcinoma (Zhang et al., 2024). They work by precisely inhibiting abnormally activated tyrosine kinases, effectively blocking tumor cell proliferation signals. BCAT can influence tumor cell resistance to TKIs by regulating metabolic reprogramming in tumor cells, affecting the utilization of BCAA and the energy supply within cells (Jiang et al., 2024).

BCAT1 has emerged as a clinically significant TKI resistance biomarker in EGFR-mutated lung cancer and is associated with shorter progression-free survival and poorer treatment outcomes (Wang et al., 2019). Zhang et al. (2024) demonstrated that BCAT1 expression is significantly elevated in TKI-resistant tumors compared with untreated tumors, highlighting its role in treatment resistance. Mechanistically, this resistance is driven by a dual metabolic–epigenetic mechanism: increased BCAA anabolic activity increases α-KG levels, promoting H3K27 demethylation and the subsequent transcriptional activation of glycolytic genes through the histone demethylases KDM6A/B, while sustained glycolytic flux supports cellular survival under TKI stress, and BCAT1 knockdown regulates the glycolytic pathway and significantly reduces the expression of key glycolytic enzymes. Importantly, inhibiting BCAT1 restores TKI sensitivity *in vivo* and inhibits tumor growth, suggesting its potential as a therapeutic target to overcome resistance. Additionally, BCAT1 is highly enriched in both mouse and human TKI-resistant chronic myeloid leukaemia (CML) cells; in a mouse model of TKI-resistant CML induced by BCR-ABL T315I, BCAT1 knockdown nearly completely eradicates leukaemia, and BCAT1 knockdown also leads to significantly reduced proliferation of TKI-resistant human leukaemia cell lines (Jiang et al., 2024). BCAT1 influences intracellular calcium levels by regulating BCAA metabolism, thereby activating the calmodulin kinase (CaMK) pathway, which ultimately leads to cAMP-response element binding protein (CREB) phosphorylation—a process that is critical for maintaining TKI-resistant CML cells. BCAT1 knockdown resulted in a significant decrease in p-CREB levels, whereas the overexpression of cAMP response element-binding protein (CREB) partially restored the proliferation of BCAT1-knockdown cells. The calcium–CaMK–CREB pathway acts downstream of the dual metabolic–epigenetic mechanism, as BCAA metabolism-driven calcium signalling is a secondary effect of metabolic reprogramming, creating a sequential regulatory cascade that sustains TKI resistance. Targeting the BCAA/BCAT1 signalling pathway or adopting a low-BCAA diet may represent potential strategies for intervening in TKI-resistant CML.

While cisplatin remains a cornerstone chemotherapeutic agent, its efficacy is frequently limited by acquired resistance. Studies have revealed that BCAT1 mediates cisplatin resistance in cervical and liver cancers through mTOR-regulated autophagy (Luo et al., 2021). Cisplatin treatment significantly upregulated BCAT1 expression at both the transcriptional and translational levels, which subsequently suppressed mTOR phosphorylation and enhanced autophagic activity, thereby reducing drug sensitivity. This resistance mechanism can be reversed through either BCAT1 knockdown or leucine supplementation, both of which restore mTOR signalling and inhibit autophagy, ultimately sensitizing cancer cells to cisplatin. Complementary research further demonstrated that BCAT1 overexpression activates autophagy-related genes and that pharmacological autophagy inhibition counteracts BCAT1-induced cisplatin resistance (Zheng et al., 2016). These findings collectively establish the BCAT1/Leu/mTOR/autophagy axis as a clinically actionable target to overcome cisplatin resistance and suggest two potential therapeutic strategies that may improve chemotherapeutic outcomes in HCC and other malignancies.

Ferroptosis is a newly identified form of cell death that emerged in the past decade and represents a highly promising target for tumor treatment. The core of ferroptosis is the overload of iron-dependent lipid peroxidation, whereas BCAT2 regulates intracellular glutamate levels, and its overexpression activates the specific antagonist system Xc, reduces lipid peroxidation levels, and decreases cell death induced by ferroptosis inducers, which suggests that high expression of BCAT2 can increase cancer cell resistance to ferroptosis-inducing agents. Notably, the ferroptosis inducers sorafenib and sulfasalazine suppress BCAT2 expression *via* ferritinophagy-mediated activation of the AMPK–SREBP1 pathway (Wang et al., 2021). Combined treatment with sorafenib and sulfasalazine significantly inhibited BCAT2 expression and increased the sensitivity of cancer cells to ferroptosis. Therefore, this study showed that BCAT2 acts as a ferroptosis inhibitor and is a potential therapeutic target for overcoming sorafenib resistance. Based on structural modelling, the T186R mutation likely alters the active site conformation of BCAT2, reducing binding affinity for inhibitors while maintaining substrate catabolism, thereby enabling tumor cells to evade targeted therapy.

### 3.2 The inhibition of BCAT resensitizes cells to endocrine therapy

Patients who are positive for oestrogen receptor α (ERα) and are receiving long-term endocrine therapy (e.g., tamoxifen and fulvestrant) may experience drug resistance. Thewes et al. (2017) found that BCAT1 expression is markedly increased in anti-oestrogen-resistant and ERα-negative breast cancers, but the limited therapeutic options available for these types of tumors represent a major clinical challenge. The long-term depletion of ERα induces BCAT1 expression in drug-resistant cells, which confirms the negative relationship between BCAT1 and ERα. Therefore, BCAT1 expression can be used as a biomarker to predict the treatment response and prognosis, and the combined use of endocrine therapy and BCAT1 inhibitors may be more effective at improving treatment outcomes.

### 3.3 The inhibition of BCAT resensitizes cells to targeted therapy

One study revealed that BCAT1-expressing IDH1-WT GBM is resistant to bevacizumab (Cho et al., 2016). In this study, bevacizumab was administered to rats, and tumor angiogenesis was assessed using DSC-perfused MRI. The results showed that both the tumor size and the nCBV significantly increased in IDH1-WT tumors treated with bevacizumab, whereas these increases were much smaller in BCAT1-knockdown tumors. Additionally, compared with BCAT1-knockdown tumors, bevacizumab-resistant IDH1-WT tumors exhibited more microvascular remodelling. The expression levels of Ki-67, CD34, and HIF-1α were significantly lower in BCAT1-knockdown tumors than in control tumors, indicating that tumor cell proliferation and angiogenesis were inhibited. These findings suggest that IDH1-WT GBMs expressing BCAT1 are more resistant to bevacizumab treatment and that the nCBV could be considered a potential alternative imaging biomarker for predicting the response to antiangiogenic therapy in GBM.

BCAT2 mutations lead to drug resistance in glioblastoma through various mechanisms, including effects on protein structure and enzymatic activity, changes in amino acid metabolic pathways, and altered regulation of signalling pathways and transcription factor activity (Mehaffey et al., 2018). BCAT2 variant sequences in glioblastoma were analysed using multistage UV photodissociation mass spectrometry (MS) and proteomic approaches. The altered kinetic properties of the BCAT2 T186R variant may contribute to drug resistance. While BCAT2 T186R could hold potential for glioblastoma management, its specific mechanisms require further investigation.

## 4 Challenges and limitations of BCAT-targeted therapy

Although preclinical evidence supporting the use of BCATs as therapeutic targets is promising, several key challenges must be addressed to translate these findings into clinical success.

A primary obstacle lies in the selectivity and specificity of inhibitors, as the currently available compounds lack sufficient isoforms selectivity, potentially leading to off-target effects on normal tissues and unintended metabolic disruption; overcoming this obstacle requires a detailed understanding of the unique active site residues and allosteric regulatory domains distinguishing BCAT1 from BCAT2 (Zhang et al., 2025). Beyond the issue of inhibitor design, tumor cells exhibit significant metabolic plasticity, enabling them to compensate for BCAT inhibition through alternative nutrient utilization pathways. For instance, the downregulation of BCAT2 in chronic lymphocytic leukaemia cells can result in upregulated BCAT1 expression, thereby maintaining BCAA catabolism and limiting therapeutic efficacy (Shao et al., 2024). Moreover, tumors may shift their dependency to glutamine or glucose metabolism, underscoring the necessity for combination strategies to block these compensatory routes.

This metabolic adaptability is compounded by the potential for toxicity to normal tissues, given that BCATs play critical roles in organs such as the brain, liver, and haematopoietic system. Inhibiting BCAT2 may disrupt mitochondrial function in healthy cells, while BCAT1 inhibition could impair immune cell function by reducing BCAA availability; therefore, clinical development must carefully balance antitumor efficacy with patient tolerability, potentially through innovations in tissue-specific drug delivery or refined dose optimization. Most existing data have been obtained from gene knockout models or *in vitro* studies, with a notable scarcity of *in vivo* pharmacokinetic and pharmacodynamic profiles for BCAT inhibitors. Compounding this problem is the current absence of validated biomarkers to identify patients most likely to benefit, highlighting the urgent need to correlate clinical outcomes with BCAT expression levels, BCAA metabolic profiles, or the activation states of downstream signalling pathways.

Future research should address these multifaceted challenges by focusing on developing isoform-specific BCAT inhibitors through structure-based drug design, investigating rational combination therapies—such as pairing a BCAT inhibitor with an mTOR inhibitor and a BCAA-restricted diet—to counteract metabolic compensation, and designing clinical trials that incorporate predictive biomarkers and pharmacodynamic endpoints to rigorously assess target engagement and the therapeutic response.

## 5 Conclusion

This review focuses on the molecular background of BCATs, their roles in various tumors, and their relationships with drug resistance in tumors, providing a multifaceted basis for tumor research and treatment. The metabolic regulatory mechanisms of BCATs in certain tumors are listed in Table 1. BCAT1 and BCAT2 exhibit distinct expression patterns, subcellular localization, regulatory mechanisms, and functional roles in various tumor types, as summarized in Table 2.

**TABLE 1:** BCAT and metabolic regulation mechanisms of certain tumors.

Tumors	BCAT expression levels	Mechanism of action	Quote
GC	BCAT1↑	Activation of PI3K/AKT/mTOR signaling pathway promotes tumor progression Positively correlates with VEGF signaling pathway and promotes tumor angiogenesis	Shu et al. (2021)
		LINC00324/miR-3200-5p/BCAT1 axis	Wang et al. (2020)
		E61A mutation, enhanced enzyme activity: enhanced BCAA catabolism leads to BCKA accumulation and activation of RhoC	Qian et al. (2023)
	BCAT2↓	Accumulation of leucine and activation of mTOR for GC growth	Wolfson et al. (2016), Zhang Y. et al. (2022)
PDAC	BCAT2↑ (in PDAC cells)	Stabilization of BCAT2 by USP1 deubiquitination at the K229 site promotes tumor cell proliferation	Li et al. (2022)
		KRAS stabilizes and promotes PDAC development by decreasing BCAT2 phosphorylation at the Y228 site and reducing TRIM21-mediated BCAT2 ubiquitination and degradation	Li et al. (2020)
		Acetylation regulation: mutants are able to stabilize BCAT2 protein and promote tumor growth	Lei et al. (2020)
		ME2 deletion combined with ME3 depletion elevates ROS levels, activates AMPK, and downregulates BCAT2 transcription	Dey et al. (2017)
	BCAT1↑ (in CAFs)	The TGF-β-SMAD5 axis directly targets BCAT1 and binds to the promoter region to promote its expression and PDAC cell growth	Zhu et al. (2020)
NSCLC	BCAT1↑	Decreased ROS content activates the NF-κB pathway and promotes LUAD progression	Yu et al. (2022)
		Regulation of c-Myc, cyclin D1 and MMP7 and upregulation of active β-catenin proteins through the Wnt/β-catenin signaling pathway	Lin et al. (2020)
		BCAT1-α-KG-miR-200c-SOX2 pathway, which promotes SOX2 expression, drives metastatic lung cancer cells	Mao et al. (2021)
		Reprogrammed BCAAs metabolism promotes α-KG-dependent demethylation of lysine 27 on H3K27, enhances glycolysis, and promotes tumor progression	Zhang et al. (2024)
		Drug resistance in EGFR mutant lung cancer cells mediated by H3K9 demethylation upregulation in STACs	Wang et al. (2019)
Leukaemia	BCAT1↑	In EZH2-deficient myeloid tumors, cooperation with NRAS G12D maintains intracellular BCAA pools, enhances mTOR signaling, and promotes the transformation of MPN to leukemia	Gu et al. (2019)
		Decreased α-KG levels and upregulation of H3K9me3 in AML cells lead to decreased DNA damage repair capacity	Pan et al. (2024)
		By regulating α-KG levels, it influences epigenetic modifications and HIF1α stability, thereby driving AML progression	Raffel et al. (2017)
		Activation of BCR/mTOR axis for CLL progression	Shao et al. (2024)
		METTL16 binds to the m6A-modified region of BCAT1/2 mRNA and promotes AML cell proliferation and self-renewal of leukemic stem cells	Han et al. (2023)
		In BC-CML, MSI2 binds to a specific sequence in the 3′-UTR of BCAT1 mRNA to stimulate mTOR activity and cancer progression	Hattori et al. (2017)
		Activation of CREB phosphorylation regulates the development of TKI-resistant CML	Jiang et al. (2024)
Glioma	BCAT1↑	HIF-1 upregulates BCAT1 expression under hypoxic conditions	Zhang et al. (2021a)
		BCAT1 promotes proliferation and invasion by downregulating α-KG, increasing HIF stability, and upregulating FOXM1 in high-expressing tumors	Fala et al. (2023)
		The lncRNA PSMB8-AS1 competitively binds to miR-382-3p to enhance BCAT1 expression and promote glioma progression	Liu et al. (2024)
		circRNA VPS18 upregulates BCAT1 expression through sponge adsorption of miR-1229-3p	Huang et al. (2022)
BC	BCAT1↑	Activation of PGC-1α, NRF-1, TFAM and β-F1-ATPase expression, activation of mTOR signaling, enhancement of citrate synthase activity, increase of cellular ATP level, reduction of mitochondrial ROS, and promotion of BC cell growth and survival	Zhang and Han (2017)
		DOT1L regulates BCAT1 expression through histone H3K79 methylation and promotes sphere formation and cell migration activity in breast cancer cell lines	Oktyabri et al. (2016)
		In TNBC, regulation of the IGF-1/insulin PI3K/Akt pathway promotes tumor survival and progression	Shafei et al. (2020)
		In TNBC, BCAT1 promotes tumorigenesis by enhancing SHOC2 expression and activation of the downstream RAS-ERK oncogenic signaling pathway	Huang et al. (2025)
HCC	BCAT1↑	BCAT1 promoter is activated by myc transcription factors, positively correlates with c-Myc, and promotes HCC cell growth and development	Xu et al. (2016), Zheng et al. (2016)
		Activation of AKT signaling pathway, induction of EMT, and promotion of HCC development	Ding et al. (2023)
CRC	BCAT1↑	TMPO-AS1/miR-98-5p/BCAT1 axis promotes CRC cell growth	Ye et al. (2022)
		Inhibition of BCAT1 expression by PSK39 contributes to improved prognosis and anti-metastasis	Yoshikawa et al. (2006)
NPC	BCAT1↑	c-Myc transcription factor directly binds to the BCAT1 gene promoter region and promotes tumor development	Zhou et al. (2013)
		FLOT2 inhibits miR-33b-5p, upregulates BCAT1 transcription *via* c-Myc, and promotes NPC cell proliferation	Liu et al. (2021)
PC	BCAT1↑	miR-218 downregulation suppress BCAT1 and leads to cellular resistance to CDDP	Zhu et al. (2017)
		Src activates BCAT1, promotes AR expression, and induces androgen-independent PC cell proliferation	Chattopadhyay et al. (2017)
	BCAT2↑	Inhibits autophagy and ferroptosis	Mei et al. (2025)
		Interacts with PCBP1 at leucine 239, regulates the PI3K/AKT signaling pathway, and promotes tumor progression	
KIRC	BCAT1↑	Drives EMT in KIRC cells by directly downregulating epithelial phenotype markers and upregulating mesenchymal phenotype markers	Li et al. (2024)
Melanoma	BCAT1↑	One of the downstream mechanisms of c-Myc reduces oxidative phosphorylation, increases glycolysis, and enhances melanoma progression	Zhang et al. (2021b)
	BCAT2↑	Regulation of FASN/ACLY expression through P300-dependent histone acetylation ZEB1 acts as an upstream transcription factor upregulated by mid-BCAT2 to promote melanoma growth	Tian et al. (2023)
EC	BCAT1↑	Activates mTORC1 signaling pathway and promotes cancer cell proliferation	Wang et al. (2018)

GC, gastric cancer; PDAC, pancreatic ductal adenocarcinoma; NSCLC, non-small cell lung cancer; LUAD, lung adenocarcinoma; AML, acute myeloid leukemia; CLL, chronic lymphocytic leukemia; MPN, myeloproliferative neoplasm; BC-CML, blast crisis chronic myeloid leukemia; TKI, tyrosine kinase inhibitor; GBM, glioblastoma; BC, breast cancer; TNBC, triple-negative breast cancer; HCC, hepatocellular carcinoma; CRC, colorectal cancer; NPC, nasopharyngeal carcinoma; PC, prostate cancer; KIRC, kidney renal clear cell carcinoma; EC, endometrial cancer; PI3K, phosphatidylinositol 3-kinase; mTOR, mammalian target of rapamycin; VEGF, vascular endothelial growth factor; BCAA, branched-chain amino acid; BCKA, branched-chain α-keto acid; RhoC, Ras homolog gene family member C; USP1, ubiquitin specific protease 1; KRAS, Kirsten rat sarcoma viral oncogene homolog; TRIM21, tripartite motif containing 21; ROS, reactive oxygen species; AMPK, AMP-activated protein kinase; TGF-β, transforming growth factor beta; CAF, cancer-associated fibroblast; NF-κB, nuclear factor kappa-light-chain-enhancer of activated B cells; MMP7, matrix metalloproteinase 7; α-KG, α-ketoglutarate; SOX2, SRY-box, transcription factor 2; EGFR, epidermal growth factor receptor; STACs, sublethal TKI-adapted cells; EZH2, enhancer of zeste homolog 2; NRAS, neuroblastoma RAS viral oncogene homolog; PARP, poly ADP-ribose polymerase; HIF-1α, hypoxia-inducible factor 1-alpha; METTL16, methyltransferase-like 16; LSC, leukemia stem cell; MSI2, Musashi RNA-binding protein 2; CREB, cAMP, response element-binding protein; FOXM1, forkhead box protein M1; lncRNA, long non-coding RNA; circRNA, circular RNA; PGC-1α, peroxisome proliferator-activated receptor-gamma co-activator-1α; NRF-1, nuclear respiratory factor-1; TFAM, mitochondrial transcription factor A; DOT1L, disruptor of telomeric silencing 1-like; IGF-1, insulin-like growth factor 1; ERK, extracellular signal-regulated kinase; EMT, epithelial-mesenchymal transition; CDDP, cisplatin; AR, androgen receptor; FASN, fatty acid synthase; ACLY, ATP-citrate lyase; ZEB1, zinc finger E-box binding homeobox 1.

**TABLE 2:** Comparison of BCAT1 and BCAT2 in tumor biology.

Feature	BCAT1	BCAT2
Localization	Gene location: Chromosome 12	Gene location: Chromosome 19
	Subcellular localization: Cytosolic (BCATc)	Subcellular localization: Mitochondrial (BCATm)
Normal tissue expression	Restricted (brain, ovary, placenta et al.)	Ubiquitous (except liver)
Expression in cancer	Upregulated: GC, PDAC (in CAFs), NSCLC, AML, CLL, BC-CML, Glioma (IDH-WT), BC, HCC, CRC, NPC, PC, KRIC, Melanoma, EC)	Upregulated: PDAC (in cancer cells), PC, Melanoma,BC
		Downregulated:GC
Primary role in BCAAs metabolism	Initial catabolism in the cytosol; may be linked to biosynthetic processes	Core mitochondrial catabolic pathway
Key regulatory mechanisms	Epigenetic: Promoter hypomethylation, H3K9/H3K27 demethylation	Post-translational modifications: Key regulation *via* ubiquitination/deubiquitination and acetylation
	Transcriptional: Direct target of c-Myc, HIF-1α, SOX2	
	Post-transcriptional: Regulated by lncRNAs, circRNAs, and miRNAs	Transcriptional: Regulated by SREBP1, ZEB1
	Oncogenic Signaling: Often upregulated by KRAS, PI3K/AKT pathways	

Cancer Types: GC, gastric cancer; PDAC, pancreatic ductal adenocarcinoma; CAFs, Cancer-Associated Fibroblasts; NSCLC, Non-Small Cell Lung Cancer; AML, acute myeloid leukemia; CLL, chronic lymphocytic leukemia; BC-CML, blast crisis chronic myelogenous leukemia; IDH-WT, Isocitrate Dehydrogenase Wild-Type; BC, breast cancer; HCC, hepatocellular carcinoma; CRC, colorectal cancer; NPC, nasopharyngeal carcinoma; PC, prostate cancer; KIRC, kidney renal clear cell carcinoma; EC, endometrial cancer; BC, Bladder Cancer. Molecular and Cellular Terms: lncRNAs, Long Non-Coding RNAs; circRNAs, Circular RNAs; miRNAs, MicroRNAs; HIF-1α, Hypoxia-Inducible Factor 1-Alpha; SOX2, SRY-Box Transcription Factor 2; KRAS, kirsten rat sarcoma viral oncogene homolog; PI3K/AKT, Phosphoinositide 3-Kinase/Protein Kinase B; SREBP1, Sterol Regulatory Element-Binding Protein 1; ZEB1, Zinc Finger E-Box Binding Homeobox 1.

BCAAs are known to improve the outcomes of patients with cirrhosis and hepatic encephalopathy (HE). Due to the protein-restricted diet of HE patients, supplementation with BCAAs can improve protein metabolism, regulate the imbalance between aromatic amino acids (AAAs) and BCAAs, reduce AAA entry into the brain, and inhibit the production of pseudoneurotransmitters (Gluud et al., 2017; Konstantis et al., 2022). Glutamine synthesis is the primary ammonia detoxification pathway in the brain, and the reactions catalysed by BCATs are involved in glutamate production and consumption. Therefore, changes in BCAT activity, particularly BCAT1 activity, may indirectly influence ammonia clearance and glutamine accumulation in the brain.

In terms of treatment, BCAT inhibitors can block BCAT enzyme activity and interfere with BCAA metabolism, thereby inhibiting tumor cell proliferation and growth or increasing the efficacy of existing therapies when combined with chemotherapy or immunotherapy. Tumor cells often have a high demand for and utilize BCAAs, and a BCAA-restricted diet can reduce the nutritional sources available to tumor cells, inhibiting their growth and proliferation (Li et al., 2022). When combined with BCAT inhibitors or other anticancer drugs, a BCAA-restricted diet may further increase treatment efficacy (Qian et al., 2023). However, the relationships between BCAT and tumors are still unclear. Although researchers currently have some understanding of the relationship between BCATs and tumors, many aspects remain unknown. While some studies have outlined the mechanisms linking BCATs to tumors, these studies lack depth in certain areas. For instance, while some studies have reported that BCATs influence metabolic pathways in certain tumors, researchers have not investigated how changes in metabolites affect the tumor microenvironment and cell–cell interactions (Boskovic et al., 2024). BCAT inhibitors have not been tested in any clinical studies to prove these conclusions. Most of these conclusions were only based on the results from small numbers of patients and not in a wider group of clinical patients.

Future studies should focus more on clarifying the mechanisms of BCATs in different tumor microenvironments and improving clinical studies on BCATs as biomarkers and therapeutic targets. Based on the mechanisms of BCATs in different tumors, the development of more specific and efficient BCAT inhibitors or modulators and thorough explorations of how to combine BCAT-targeted therapies with existing tumor treatment regimens (such as surgery, radiotherapy, and chemotherapy) can provide guidance for the development of comprehensive treatment strategies in clinical practice, thereby advancing the development of precision cancer therapy.
